# Reassessing Fitness-to-Drive in Drinker Drivers: The Role of Cognition and Personality

**DOI:** 10.3390/ijerph182312828

**Published:** 2021-12-05

**Authors:** Luigi Tinella, Alessandro Oronzo Caffò, Antonella Lopez, Francesco Nardulli, Ignazio Grattagliano, Andrea Bosco

**Affiliations:** 1 Department of Educational Sciences, Psychology, Communication, University of Bari, 70121 Bari, Italy; alessandro.caffo@uniba.it (A.O.C.); antonella.lopez@uniba.it (A.L.); ignazio.grattagliano@uniba.it (I.G.); andrea.bosco@uniba.it (A.B.); 2 Commissione Medica Locale Patenti Speciali, Azienda Sanitaria Locale-Bari, 70123 Bari, Italy; francesco.nardulli@asl.bari.it

**Keywords:** drunk driving, fitness-to-drive, cognition, spatial transformation skills, personality

## Abstract

Drunken driving is among the main challenges for road safety by causing worldwide motor-vehicle crashes with severe injuries and deaths. The reassessment of fitness-to-drive in drivers stopped for drunken driving includes mainly psychological examinations. The present study aimed to investigate the effectiveness and the consistency of selected variables of different psychological driving-related dimensions (i.e., cognitive skills and personality) in discriminating 90 male drinker drivers (DD) from matched non-drinkers controls. The Montreal Cognitive Assessment (MoCA), the Mental Rotation Test (MRT), and the Perspective-Taking Test (PT) were administered to assess overall cognitive functioning, and object- and self-based spatial transformation abilities, respectively. Participants completed a computerized test measuring resilience of attention (DT), reaction times (RT), and perceptual speed (ATAVT). The Personality Psychopathology Five scales (i.e., PSY-5: Aggressiveness-AGGR, Psychoticism-PSYC, Disconstraint-DISC, Negative-Emotionality-NEGE, and Introversion-INTR) the validity scale (L) and the dissimulation index (F-K) were scored from the Minnesota Multiphasic Personality Inventory (MMPI-2). A logistic binomial regression analysis (backward subtraction method) was used to identify discriminant predictors. A prediction analysis (ROC curve method) was performed on the final model. Results showed that the scores obtained in MRT, DT, and the personality measures of PSYC, DISC, NEGE, and INTR significantly discriminated DD from their matched controls with moderate-to-good values of accuracy (0.79), sensitivity (0.80), and specificity (0.79), as well as a good AUC value (0.89). In some cases, the personality dimensions provided—reliable—unexpected results. Low scores of PSYC, NEGE, and INTR were found to predict the membership to the DD group; results are discussed with reference to response management. Personality measures should be assessed with particular attention in a forensic context because they are more prone to be feigned than cognitive ones. Overall, the present study confirmed the relevance of integrating different driving-related psychological dimensions in the evaluation of fitness-to-drive showing the usefulness of standardized tools for the reassessment of drinker drivers.

## 1. Introduction

Drunken driving represents one of the most important issues for road safety [1,2]. Alcohol affects the ability to safely manage the vehicle, to stay in the lane and to maintain a constant speed, also impairing the driver’s concentration and all aspects of driving safety as, for example, the tendency towards risks [3,4,5]. Worldwide, driving under the influence of alcohol and other substances is among the most frequent causes of motor-vehicle crashes [6]. Despite the great efforts of institutions in preventing drunk driving, it still can be considered as the most relevant cause of serious injury or death behind the wheel [1].

During 2019, in the United States there was a fatality every 52 min in a drunk driving crash [1]. In the same year, Italy registered an average of 55 road fatalities per million inhabitants, above average with respect to the other countries of the European Union [7]. A nationwide survey in Italy revealed an increase in citations for drunken driving from 2018. The total number of police-recorded crashes with severe injuries was equal to 58.872 and those with at least one drunk driver were 5.117, constituting 8.7% of the total [7]. This framework clarifies the detrimental consequences of alcohol consumption on driving safety. In the Italian context, people stopped for driving under the influence of alcohol undergo from expensive pecuniary sanction to the driving license suspension, recoverable only by submitting to a reassessment of fitness to drive (art.186 of the Italian Traffic Laws—“Nuovo codice della strada”).

### 1.1. Alcohol, Cognitive Functioning, and Driving Performance

Many studies have focused on the effects of alcohol on both cognition and driving performance [8,9]. Regarding cognition, the alcohol assumption is associated with a decrease in psychomotor speed, and having an influence on working memory capacity, divided attention, visual acuity, and perceptual accuracy and speed [3,8,10]. Some scholars have posited that habitual alcohol consumption mainly causes a restriction of the attentional focus to most salient environmental cues only, leading to a decreased attentional processing (i.e., the attentional-allocation model) [11,12]. Another theoretical approach (i.e., the impaired response inhibition model) suggested that alcohol affects mainly the response inhibition rather than the attention focus, leading to impaired inhibition processes of behavioral activation [13]. Bartholow et al. [8] demonstrated that alcohol affects the response selection accuracy more than attentional processes in terms of times. Thus, alcohol influences more the selection of the correct response between competitive alternatives than the processing speed, leading to negative effects on the response accuracy more than on the response time [10,13,14]. Therefore, alcohol-related changes in driving performance may reflect these attentional restrictions with negative effects on road safety [15]. Frontal/executive functions are also related to the etiology of alcohol-related problems [16]. Executive function’ deficits are risk factors for the development of alcohol-related disorders, and make drinkers more exposed to experience problems as a consequence of the assumption [17,18].

Measures of overall functioning also have shown to be associated with driving performance of young [19,20], adult [20,21] and old drivers [19,22]. In particular, the score in both the Montreal Cognitive Assessment (MoCA) [23] and the Mini-Mental State Examination (MMSE) [24], two of the most widely used tools of mental status in older adults [25], was found to significantly predict measures of both fitness-to-drive cognitive prerequisites [21,26] and simulated driving performance [19]. The MoCA has been shown as a useful tool for detecting alcohol-related cognitive changes [27,28] and, in addition to measures of physical frailty, it has been shown to predict the hospitalization for neurological problems in people with alcoholic liver disease [29].

Among cognitive domains spatial cognition is generally regarded as a domain highly affected by the consumption of alcohol [30]. Measures of visuospatial learning, perception, construction, and memory have been shown to discriminate alcoholic groups from matched controls, while measures of spatial knowledge discriminated alcoholics with multiple other drugs from those who abuse alcohol and marijuana or alcohol only [30]. It was demonstrated that representations and transformations of visuospatial information play a key role in supporting driving performance and spatial cognitive style predicts driving behavior [31,32,33]. Recently, measures of object-and self-based spatial transformation skills, evaluated through Mental Rotation and Perspective-Taking, respectively, have been found to predict fitness-to-drive in young and old-adult drivers, and to mediate the effects of global cognition on both perceptual speed and resilience of attention to traffic stress [20,26]. Spatial transformation skills are involved in driving activity, supporting the execution of driving maneuvers and they are sensitive to alcohol-related changes in cognitive processing [30].

### 1.2. The Relationship between Drunk Driving and Driving-Related Personality Traits

Alcohol assumption is associated not only with cognition and performance but also with dispositional factors (e.g., judgment, aggressiveness, and impulsivity) which, in turn, were found to be related to driving violations and increased crash risk [34,35].

Early studies on personality showed that impulsivity [36], aggressiveness, sensation-seeking, antisocial behavior, extraversion, and hostility characterize drinker drivers. Among these, sensation-seeking was shown as the personality trait with the most consistent predictive validity on driving measures [37,38,39,40]. Higher sensation seekers were more likely to drive after drinking alcohol, without wearing safety belts, and show low-risk aversion [41,42,43]. The last decades have witnessed increase in studies focused on the driver’s attitude toward drink-and-drive, behavioral habits, and values as more strictly determinants of drunken driving suggesting that personality is not enough to predict the drinker driver behavioral pattern. Previous studies [44] have demonstrated that a positive vision about the effects of alcohol, a low social desirability, and frequent drinking were stricter predictors of drinker driver behaviors than personality traits. Attitudes mediated the relationship between personality and risky driving, showing to be a useful self-reported measure in predicting recidivism [34,45].

Recently, few studies investigated the role of pathological personality traits in the evaluation of psychological fitness to drive [40,46,47].

It has been demonstrated that psychopathological personality indicators (PSY-5 scales) included in the Minnesota Multiphasic Personality Inventory-2 (MMPI-2) [48] could be useful in the screening of the personality profile of drivers predicting their traffic stress resilience and reaction times [40].

Following the PSY-5 model, the personality traits of Aggressiveness (AGGR), Psychoticism (PSYC), Disconstraint (DISC), Negative-Emotionality/Neuroticism (NEGE), and Introversion (INTR) constitutes five broad traits underlying individual differences that could impact clinical problems [48]. Aggressiveness refers to the use of anger and aggression to alarm others and to achieve aims [49]. Psychoticism refers to detachment from reality with alienation while Disconstraint refers to the entity to which behavior is related to future consequences [47]. Introversion reflects how much respondents are focused on their own thoughts, feelings, and mood while Negative-emotionality is a measure of anxiety and sensitivity to the danger [47]. Strictly associated with sensation-seeking, Disconstraint is indicative of neuro-behavioral disinhibition and is related to the persistence of alcohol use and abuse [50]. Together with Neuroticism, Disconstraint is associated with aspects of addictive behaviors [51,52,53]. On the other hand, Introversion seems inversely related to road crashes and aberrant driving behaviors [52] while it was found to predict motor speed in young and adult drivers [40].

Despite the PSY-5 scales having been shown as useful measures able to detect alcohol-associated personality problems, their usefulness in assessing the drinker drivers’ personality for relicensing purposes has not been previously investigated.

Furthermore, it is known that self-reported measures of personality are particularly affected by response management [54]. During a forensic evaluation for the reassessment of fitness to drive, people tend to dissimulate undesirable aspects of their behaviors. This probably is associated to the face validity of scales; that is, the extent to which the content of items appears obvious and clearly recognizable.

With reference to the scales of the MMPI-2 the impression management was found to significantly affect responses to both the basic clinical scales, the content scales, and the PSY-5 scales in participants who underwent to a parental suitability assessment [55,56]. The validity scales of the MMPI were introduced to detect the acceptability of the protocol for the interpretation [57]. Moreover, these scales provide qualitative information on the response’s distortion. For example, the Lie (L) scale detect the respondent’s attempt to be defensive [58]; high scores in L scale point out the tendency to positively manipulate the self-image during an assessment [59]. The F-K index or ‘Gough index of dissimulation’ [60], the score difference between the validity scales F and K, is considered a measure of both defensiveness and malingering [58]. While a high F-K index indicates the respondent’s tendency to malinger by overreporting psychopathology [58,61,62], a low index suggests a defensive profile underreporting psychopathology [56,58,61].

What happens is that in the context of fitness-to-drive reassessment, it is likely that respondents are highly motivated to both provide a positive self-image in self-reported questionnaire [63,64] and show their maximum performance in cognitive and driving tasks. While self-reported data could be considered potentially and intrinsically affected by faking good, the performance in cognitive and driving tasks is less exposed to this bias. To date, it is unclear which driving-related measures are most affected by the response management of drinker drivers, and which psychopathological personality traits are mostly affected by response manipulations in these samples.

### 1.3. The Assessment of Fitness-to-Drive

Different evaluation tools have different sensitivity in detecting alcohol-related changes in driving performance [10]. The sensitivity to the effect of alcohol is greater if evaluated through driving performance tests (e.g., Driving Simulation—DS; On-Road driving Tests—ORT) and tasks of controlled performance (i.e., oculomotor coordination) than in tests evaluating the automatic performance (i.e., simple and choice reaction tests) [10].

The ORT, the DS, and measures of divided attention seems to be highly sensitive to the estimation of alcohol-related impairment at low doses [65]; measures of visuospatial functioning, visual tracking, perception, and cognitive tests are only sensitive to high doses [10,65,66]. In their review, Jongen et al. [10] concluded that go/no-go tasks and measures of divided attention are highly sensitive at medium and high blood alcohol concentration levels, also showing the consistent sensitivity of driving performance tests at medium levels. Overall, the ORT showed to be more sensitive in detecting alcohol impairments when compared to DS assessments, which, in turn, suffer a lack of shared protocols and standardized measures [66]. Anyway, according to authors, limited information is currently provided in terms of which specific laboratory tasks should be included for the identification of drinker drivers in the assessment of driving fitness. This knowledge gap assumes greater importance considering that drivers who attend a relicensing assessment are evaluated in a sober state. One of the aims of the assessment is to detect patterns of alcohol-related cognitive signs associated to the likelihood that the driver gets behind the wheel after drinking.

Laboratory tests are a cost-effective standardized screening in assessing driving behavior as well as fitness-to-drive [67,68] and have been shown to be able in detecting alcohol related behavioral signs demonstrating consistent reliability across studies [69,70]. The laboratory assessment of fitness-to-drive includes the computerized evaluation of its cognitive prerequisites (i.e., Alertness, Attention, Simple and Choice reaction, Visual perception, Perceptual speed, etc.). These instruments provide several advantages because of their low cost, handling, standardization, and predictive validity on both simulated and on-road naturalistic driving. For example, it was demonstrated that the Motor-Free Visual Perception Test (MVPT-revised) [69], developed as a valid measure of visual processing skills, showed high predictive validity on driving skills and it was associated to highway safety and on-road driving, with good sensitivity to the effects of alcohol [71,72,73,74]. The DRIVESC package of the Wien Test System is a computerized screening task evaluating the resilience of attention, reaction times, and perceptual speed. This was recently used for identifying psychological fitness-to-drive among intoxicated drivers [73]. In their study, Nechtelberger et al. [73] demonstrated that the driving-related cognitive prerequisites were influenced by age and measures of fluid intelligence in drivers caught under the effect of alcohol or drugs. Authors showed also that driving related personality traits (i.e., emotional instability, self-control, and the sense of responsibility) contributed to explaining the performance [73].

Overall, literature on the assessment of psychological fitness-to-drive seems to suggest that a multi-dimensional approach including cognitive, behavioral, and personality evaluation is needed to achieve more accuracy for both relicensing and prevention. Moreover, it remains unclear if a comprehensive protocol of evaluation of the psychological fitness-to-drive could be able to identify personality and cognitive patterns associated to drinker drivers far away by the intoxication episode.

### 1.4. The Present Study

As stated above, a great part of the literature on the psychological evaluation of drinker drivers was mainly focused on the study of both the long-term effects of alcohol on driving measures and on the impaired driving performance under the effects of alcohol.

Conversely, more studies should be devoted to investigating cognitive and personality signs of drinker drivers assessed in a sober state.

The general objective of this study was to investigate cognitive and personality characteristics associated to drinker drivers (DD), already stopped for drunken driving, comparing them with non-drinker drivers paired for age and education. In particular, the study assessed the usefulness of a selected pool of cognitive, behavioral, and personality measures in discriminating DD from matched non-drinkers controls. Measures of both cognitive functioning (MoCA) and spatial transformation abilities (i.e., Mental Rotation and Perspective Taking) were taken as cognitive factors. Driving skills were assessed by using the DRIVESC package of the Wien Test System [74] considering resilience of attention, reaction speed, motor speed, and perceptual speed, separately. The Lie scale of validity (L) and the Gough dissimulation index (F-K) were scored from the validity scales of the MMPI-2 questionnaire. Finally, personality traits from the PSY-Five model [75] (i.e., Aggressiveness—AGGR, Psychoticism—PSYC, Disconstraint—DISC, Negative-Emotionality—NEGE, and Introversion—INTR) were considered as personality predictors.

The study was also aimed to test the hypothesis that different psychological measures could differentially discriminate the DD fitness-to-drive depending on different manageability of responses and performances. It was hypothesized that performance measures would have showed higher efficiency in discriminating groups than self-reported ones. Among cognitive measures, complex cognitive tasks, requiring high processing effort such as imagined spatial transformations, were expected to show higher discriminative capability rather than measures of mental status which in turn require less cognitive effort. Moreover, following the classification of driving-related human factors given by Elander et al., [76] intrinsic factors to driving; that is, driving skills (i.e., in our study: resilience of attention, reaction times, and perceptual speed) are expected to be highly sensitive in discriminating DD. Measures of cognition (i.e., MoCA, MRT, and OPT) and personality (AGGR; PSYC, DISC, NEGE, and INTR), which instead were classified as factors extrinsic to driving by Elander et al. [76], are expected to be less sensitive in discriminating the group membership with differences among scales. Considering measures of cognition, both the overall functioning and the spatial transformation skills were previously shown to be sensitive to both the driving capability and the effects of alcohol [26,27,29]. Considering personality scales, AGGR [77], DISC, and NEGE [51,52] were expected to characterize more the DD group than the control group which in turn was expected to show higher scores of INTR [53]. No effects were expected for the PSYC personality trait. Finally, the Lie scale and the tendency to dissimulation were expected to be higher in DD participants than in controls. Table S1 resumes the expected discriminatory capability for cognitive measures, driving skills, and personality traits.

## 2. Materials and Methods

### 2.1. Participants

According to the Italian traffic laws, drivers stopped for drunk driving with a Blood Alcohol Concentration (BAC) level between 0.8 and 1.5 g/l are subjected to a driving license revision through a deep medical and psychological assessment carried on by an in-charge medical committee. Ninety offenders—all males—(age M ± SD = 38.2 ± 13.6; level of education M ± SD = 11.3 ± 3.54, years) stopped for the first time for drunk driving by the police, were consecutively recruited in the study between May 2019 and December 2020 with the help of the local medical committee of Bari, Italy (Commissione Medica Locale—ASL Bari). During the recruitment period, no females were required to be relicensed. Participants in this group were tested after a month of sobriety (i.e., submitting clinical blood tests) from the intoxication event. As stated by McKnight and colleagues [78] people have been stopped driving under the influence of alcohol, can be considered frequent alcohol consumer, and in turn, drinker drivers (DD).

Participants were matched with ninety male volunteer non-drinker drivers; all were Italian active drivers paired for age and level of education (i.e., age M ± SD = 38.2 ± 17.9; level of education M ± SD = 11.7 ± 2.64). Control participants were enrolled with the support of a proxy informant and matched by using the Propensity Score Matching (PSM), performed with ‘matching’ package of R software [79]. They were required: (i) to hold a valid current driving license; (ii) to have normal or corrected vision; (iii) have driven more than one time within the last month; (iv) to be teetotal; (v) have never been sanctioned for drunk driving, and (vi) not to be or to have been in the past a professional driver. All participants had a car driving license and the years of license were almost equal for the two groups (DD: M ± SD = 19.4 ± 13.2; Controls: M ± SD = 19.7 ± 16.2).

All participants signed their informed consent before the enrolment in the study. The Ethical Committee of the Department of Education, Psychology, and Communication of the University of Bari approved the study protocol, and the whole study was performed following the Helsinki Declaration and its later amendments.

The final sample size far exceeded the criterion of a 10:1 ratio between participants and independent variables included in the model [80], and it was considered adequate to warrant the correct rejection of the null hypothesis.

### 2.2. Materials and Procedures

All participants were in a good general state of physical and psychological health at the time of the assessment session. The participants in both groups were subjected to the same experimental protocol comprising demographic questionnaire, a cognitive evaluation, a computerized evaluation of the fitness-to-drive, and a personality questionnaire. A brief interview was administered by supervised trainees in clinical neuropsychology (i) to collect demographic information; (ii) to exclude neurodegenerative and vision/acoustics disorders, and (iii) to gather information about participant’s driving rates. After completing the interview, all participants completed the below-described tests.

#### 2.2.1. Cognitive Functioning

The Montreal Cognitive Assessment (MoCA) [23] was administered to evaluate participants mental status. The MoCA briefly assess a wide range of cognitive domains including visuospatial/executive, naming, attention, language, abstraction, memory, and orientation. Raw scores are reported on a 30-point scale and one adjunctive point is provided for those participants with <12 years of education. A cut-off score = 17 was found as the best one for discriminating participants with probable cognitive impairment in an Italian sample [81,82].

#### 2.2.2. Spatial Transformation Skills

To assess participants’ spatial transformation skills both the Mental Rotation Test (MRT) [83] and the Object-Perspective Taking Test (OPT) [84] were administered. The MRT, a measure of object-based spatial transformation, is composed of 20 items (i.e., two-dimensional drawings of tri-dimensional figures) that includes a criterion figure and four response options (i.e., two correct and two distractors). Correct alternatives have a rotated structure but identical to the criterion. Participants must find the two figures out of four that represented rotations of the criterion. The test is divided into two parts, and 3 min are provided to accomplish each part. The entire procedure takes about 10 min.

The OPT [85] provide a measure of self-based spatial transformation skills. It is composed of 12 items that shows a configuration of seven objects drawn on the top half of the sheet paper. Participants have to imagine being at the position of one object (A), facing another object (B), and to indicate the direction to a third object (C; the target). In the bottom half of the sheet paper, the imagined standing point (A) was in the center of a drawn circle while the imagined heading point (B) was represented by an arrow pointing vertically up. Participants have to draw an arrow from the center of the circle pointing the direction of the target (C). The item score is the absolute directional error that is the deviation in degrees between the observed answer and the correct direction to the target. The total score is the average deviation across items. High total scores indicate lower level of ability in OPT. The time limit to accomplish the test was 5 min. The entire procedure takes about 10 min.

#### 2.2.3. Fitness-to-Drive Screening

The cognitive prerequisites for fitness-to-drive were assessed by using the Drivesc package of the Vienna Test System [74]. It is a computerized test divided into three subtasks which evaluates the resilience of attention (Determination Test; DT), reaction times (Reaction Speed: RS and Motor Speed: MS) and the perceptual speed (Adaptive Tachistoscopic Traffic Perception Test (ATAVT), respectively. The apparatus includes an ergonomic response panel, foot pedals, a standard audio output device (headset), and a video screen. The experimental screening took approximately 25 min.

The RT involves the ability to respond as quickly and accurately as possible to specific auditory and visual stimuli. It provides two distinct measures, namely Reaction speed (i.e., the time taken to initiate the physical movement) and Motor speed (i.e., the time between the moment in which the participant’s finger leaves the rest button and the moment in which the reaction button is pressed). Times are taped in milliseconds: a short time of reaction (i.e., high visual and motor reaction speed) corresponded to a higher ability to quickly respond.

The DT is a measure of resilience of attention. Participants must react as quickly and accurately as possible to changing acoustic and visual stimuli different for frequency and colors, respectively. The software varies the speed of stimuli presentation, through a computer adaptive system, based on the respondent’s ongoing performance in terms of accuracy (i.e., hits, omissions, and false alarms) and response delay (i.e., milliseconds), providing a unique score.

The ATAVT measures the ability to quickly gain an overview of the traffic scenario. This subtask was administered in the right-hand traffic form according to the Italian Traffic Laws. Pictures of traffic scenarios were presented very briefly after an acoustic cue. After each picture, the participants must select which one, or more, objects out of a provided list of five (i.e., motorcycles/bicycles, automobiles, traffic signs, traffic lights, and pedestrians) they have perceived. The total score is the number of correct responses (omissions and false alarms are also recorded).

The total scores of the three subtasks were reported as percentile ranks with higher scores indicating a better performance. The entire procedure was made clear to the participants beforehand. All participants were assessed individually in a silent and well-lit room, without disturbances. Participants in the experimental group were assessed at the mobility center of the city while those in the control group in the Department of Psychology of the University with the same apparatus. Each assessment session was accomplished by instructed research assistants and lasted 60–90 min, with breaks provided as requested by participants.

#### 2.2.4. Personality Assessment

The Personality Psychopathology Five traits were scored from the entire pool of items of the Minnesota Multiphasic Personality Inventory-2 (MMPI-2). The MMPI-2 [86] is a 567-items questionnaire widely used for the evaluation of the personality profile. It includes true-or-false answers. Scores of personality scales are reported as T-scores. Following the Italian validation of the MMPI-2 [87], a T-score equal or greater than 65 represented a clinically relevant score. The PSY-5 scale includes traits of aggressiveness (AGGR), psychoticism (PSYC), constraint (DISC), negative emotionality/neuroticism (NEGE), and introversion (INTR). According to the Personality Psychopathology Five model [48], these five broad traits are relevant in everyone’s daily living and describe individual differences that could impact clinical problems. The Lie scale of validity (L) was reported as a T-score. The “Gough dissimulation index” (F-K) was obtained by the raw score difference between the validity scales F and K.

### 2.3. Statistical Analysis

The data were analyzed using SPSS 21.0 [88] and Jamovi 1.0.7 [89] statistical software. Two steps of analysis were performed: (a) preliminary descriptive and univariate mean comparisons, and (b) binomial regression. The first step of the analysis was aimed to clarify the univariate relationships between the criterion (i.e., people stopped for DD vs. control) and the predictors (selected measures of cognition: MoCA, MRT, OPT; personality: PSY-5 scales, Lie scale, dissimulation index; and fitness-to-drive: DT, RS, MS, ATAVT) of the subsequent logistic multiple regression. Backward stepwise method was used to minimize the number of relevant predictors in classifying group membership (out if criterion: *p* > 0.1). The remaining predictors were then entered into a final predictive model according to following formula:

*Ln (odds of Y*) = *a* + *b*1 .*x*1 + *b*2 .*x*2 + *b*3 .*x*3 … + *bn* .*x*,

where *Y* is the dependent variable (i.e., group membership), *a* is a constant, *b* are the regression coefficients and the *x* are the independent variables (i.e., psychological indicators). For a certain score obtained in each measure, the probability of belonging to the group of drivers stopped for drunken driving can be obtained considering the following formula:

$$p\;\left(x;b,w\right)=\frac{1}{1+e^{-\left(a+x1B1+x2B2+x3B3\ldots +xnBn\right)}{}^{}}$$

by replacing the *x* of the predictor (*B*) with the score obtained in the corresponding test/scale. The term *a* indicates the intercept of the regression, all *B*’s refer to regression coefficients, while the *e* represents the Euler’s number. Minimizing the misclassification rate, one should predict *Y* = 1 when *p* ≥ 0.5 and *Y* = 0 when *p* < 0.5. The accuracy of the model was assessed by calculating the area under the Receiver Operating Characteristics Curve (ROC curve; area under curve—AUC) which indicates the relative sensitivity and specificity of the model.

## 3. Results

### 3.1. Descriptive Statistics and Preliminary Analyses

Means, standard deviations, correlation coefficients, and reliability measures for the variables employed in the study are shown in the Supplementary Table S2. All participants included in the study showed a valid and interpretable personality profile (T-scores: L < 65; F < 65). Negative associations were found between age and measures of overall cognition, spatial transformation skills, and all the observed driving prerequisites. Age was negatively associated with personality measures of DISC, in both groups. The increase in age was associated with a reduction in performance. Cognitive measures (i.e., MoCA, MRT, and OPT) were found positively correlated within them and with driving measures. Measures of DISC and INTR were positively associated with the resilience of attention and reaction speed, respectively.

The results of t-tests revealed significant differences between the two groups (i.e., DD and controls) in the MRT performance (i.e., Welch’s-t(173) = 3.21, *p* < 0.01, d = 0.480), and in RS performance (i.e., t(178) = −2.60, *p* < 0.01, d = 0.389) as well as in some personality traits (i.e., PSYC: t(178) = 3.65, *p* < 0.001, d = 0.545; DISC: t(169) = −3.49, *p* < 0.001, d = −0.521; NEGE: t(178) = 7.07, *p* < 0.001, d = 1.54; and INTR: t(178) = 6.07, *p* < 0.001, d = 0.908; F-K: t(178) = 2.31, *p* = 0.022). Participants in the DD group performed significantly worse at the MRT than those in the control group, showing also lower RS, lower scores of NEGE and INTR, higher scores of DISC, and a higher tendency to dissimulate. No significant difference was found between groups considering the other employed variables (i.e., age, level of education, MoCA, OPT, DT, MS, ATAVT, AGGR, and L). The years of driving license were not significantly different between the two groups (t(178) = 0.180, *p* < 0.857, d = 0.026).

### 3.2. Binomial Logistic Regression and ROC Curves

First, all the independent variables were entered as cognitive (i.e., MoCA, MRT, and OPT), driving ability (i.e., DT, RS, MS, and ATAVT), and personality (i.e., AGGR, PSYC, DISC, NEGE, INTR, L, and F-K) predictors of the group variable. The initial model with all the 14 predictors had a -2LL of 139.325, a Nagelkerke R^2^ of 0.611, and classified 80.6% of cases correctly. After eight backward stepwise iterations the final model included seven predictors (Table 1), had a -2LL of 143.672, a Nagelkerke R^2^ of 0.593, and correctly classified 79.4% of cases. Except for MoCA, significant results were found for the effects of all predictors retained in the final model (i.e., MRT; DT; PSYC; DISC; NEGE; and INTR) and reported in Table 1.

**Figure 1:**
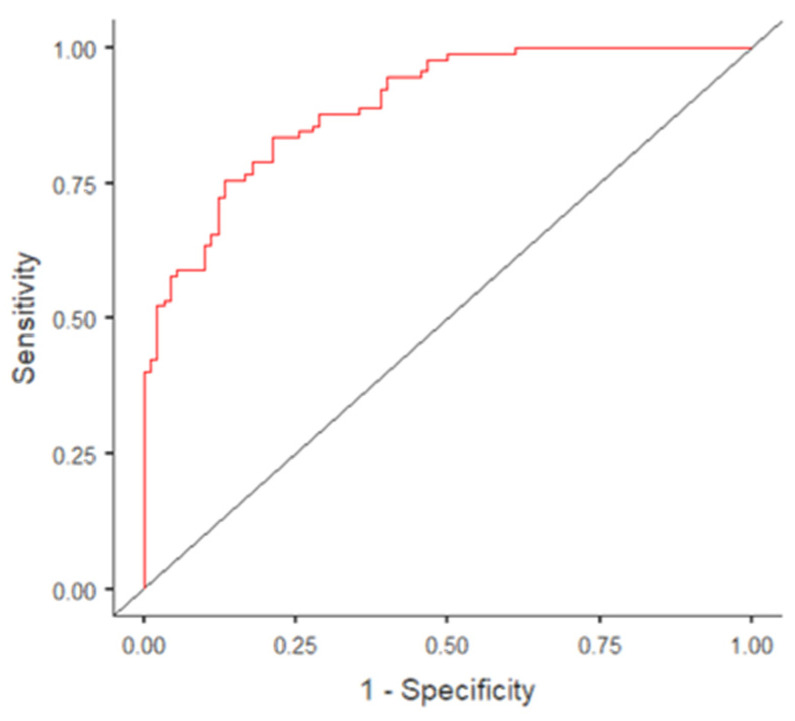
The ROC curve for the final predictive model. ROC indicates receiver-operator characteristics.

According to the aim of the study, the evaluation of the potential usefulness of the final model of variables in differentiating people at their first stop for drunk driving from non-stopped controls was carried out. The AUC value of the final model was 0.89, with sensitivity equal to 0.80, specificity equal to 0.79, and an accuracy of 0.79. The ROC curve is reported in Figure 1. The result suggested the effectiveness of the tested protocol of assessment in detecting DD drivers.

## 4. Discussion

This study aimed to detect distinctive cognitive and personality characteristics of drivers caught for drunk driving able in discriminating them from non-drinker driver controls. The effectiveness and consistency of a selected pool of cognitive, behavioral, and personality driving-related variables was tested in discriminating male drivers stopped for drunk driving from paired controls by evaluating its sensitivity and specificity in classifying drivers as belonging to one of the two groups.

Differences between participants’ groups were found in measures of reaction speed, mental rotation, psychoticism, disconstraint, negative-emotionality, introversion, and in the dissimulation index. DD participants showed lower reaction speed and mental rotation ability than controls also reporting a lower level of Psychoticism, Negative-Emotionality, and Introversion. Moreover, DD participants showed a higher level of disconstraint and a greater tendency to dissimulate than controls. Based on the binomial logistic regression analysis, measures of mental rotation, the resilience of attention, and the personality measures of Psychoticism, Disconstraint, Negative-Emotionality, and Introversion significantly discriminated drivers stopped for drunken driving from their matched controls with adequate values of both sensitivity and specificity. Measures of overall cognitive functioning, evaluated with the MoCA, showed a non-significant effect in predicting the group membership. Anyway, this measure was retained in the final predictive model contributing to the explained variance. Considering that DD participants were assessed in a sober state this result suggests that cognitive measure assessed with the MoCA is not particularly sensitive to detect differences in cognition in the two groups.

In particular, the final model showed moderate-to-good values of accuracy (0.79), sensitivity (0.80) and specificity (0.79) in predicting groups membership, as well as a very good AUC value (0.89), and a number of correct classifications, slightly under 80%. No significant results were found for the effect of perspective-taking skills, the remaining driving skills (reaction, motor, and perceptual speed), and personality scales (AGGR, L, F-K).

The reassessment of fitness-to-drive in DD represents an important issue for road safety. Previous studies focused on the usefulness of laboratory testing for the assessment of both the long-term effects of alcohol consumption on driving performance and the driving performance under the effects of alcohol. These studies identified how cognitive functions, and personality traits were influenced by the effects of alcohol behind the wheel. Less is known on which specific cognitive and personality characteristics are associated with drinker drivers and are able in discriminating them in a sober state from non-drinker drivers. The effort of the present work was oriented to provide a comprehensive psychological profile of the DD caught for drunken driving. For this purpose, the study was conducted including a sample of drivers at their first stop for drunken driving, which was evaluated in a sober state and compared to non-drinker matched controls. Overall, the present study showed the clinical usefulness of selected psychological (i.e., cognitive, behavioral, and personality) variables in discriminating DD from matched controls. An application of the logistic regression formula based on the results of the present study is provided to estimate the probability to belong to one of the two groups (supplementary File S3). The interested reader should consider that the estimation is suitable (and should be taken with caution) for male drivers aged from 18 to 79 with a schooling ranging from 5 to 24.

### 4.1. Mental Rotation

Mental rotation skills showed to significantly predict the likelihood of group membership among participants. The group of DD performed significantly worse than their paired controls. While previous studies found no significant effects of alcohol assumption on the mental rotation ability [90], more recent studies have shown alcohol-related changes in the neural activation underlying the execution of rotation tasks [70]. Considering the results presented here, the worse mental rotation performance of DD compared with controls is supposed to be (1) the effect on performance of stable changes in the brain for the effect of a persistent alcohol use or (2) the effect of associations between a poor mental rotation ability (no alcohol-related) with the assumption of maladaptive behaviors such as driving in a state of intoxication. The last assumption implies common cognitive deficits relating to poor mental rotation abilities with the likelihood of driving after drinking and being caught for drunken driving. Working memory is considered a relevant executive function associated with mental rotation skills [91,92]. Moreover, a poor working memory capability together with reduced inhibition was found associated with the relapse after a period of sobriety in people whit alcohol-related disorders [93]. It seems likely that the relationship between poor mental rotation abilities and the membership to the DD group is influenced by executive functions deficits that make the driver less efficient in mentally manipulating objects and visual scenes as well as being prone to driving violations. This explanation was supported by the significant effect of both resilience of attention (DT) and Disconstraint (DISC) among predictors, which involve measures of response inhibition and both attentional and behavioral control. Such pattern of associations deserves further and more detailed investigation.

### 4.2. Resilience of Attention

The present study showed the significant—weak—effect of the resilience of attention in discriminating DD from controls. The determination test is a complex reaction task in which irritating feelings and psychological stress are elicited, providing a measure of behavioral control [74]. According to Jongen et al. [68] this task is often used to assess the pharmacological and drug-induced driving impairment. Among the set of cognitive-motor prerequisites for fitness-to-drive, the resilience of attention to traffic stress was the one that remains as significant predictor in the final model. The complex interrelation between stress vulnerability and alcohol consumption and craving has been deepen investigated highlighting bidirectional influences [94]. An excessive alcohol use was found to be triggered by craving in reaction to stress after an abstinence period [95]. Previous studies have demonstrated that both early life adversities [96,97] and the experimental-induced stress are associated with alcohol-related behaviors [98] by showing the influence of past, chronic, and acute stress on both alcohol seeking and consumption. This evidence shows that a greater vulnerability to stress may be the cause of alcohol seeking and consumption and may suggest, together with results of the present study, the key role of a low resilience to stress. Conversely, chronic-alcohol consumption may be considered as a stressor by activating the hypothalamic–pituitary–adrenal axis (HPA) [99] and neuroadaptation with consequences on cognitive and behavioral control processes.

This result also provide support for the evidence on the association between executive functions deficits and alcohol-related behaviors [17,18]. According to Day et al. [16] the exact links by which this occurs are not completely clear. In any event, deficits in set shifting, updating, and inhibition may respectively influence both the ability to engage in coping strategies to deal with alcohol-related stimuli and the ability to resist to engage in drinking behaviors [16]. The result presented here extends this evidence to risky driving behaviors.

Finally, the result presented here confirms that measures of complex performance are stronger predictors of fitness-to-drive compared with measures of simple-reaction [100], extending this evidence to the evaluation of fitness-to-drive in DD drivers.

### 4.3. Personality Traits

Four out of five personality scales showed a significant effect in the final predictive model: Psychoticism, Disconstraint, Negative-Emotionality, and Introversion. No significant result was found for the effects of both the Lie scale and the dissimulation index.

Results showed that participant who reported higher scores in Disconstraint, and lower scores in Psychoticism, Negative-emotionality, and Introversion were more likely to be classified in the group of DD. The association between the personality trait of Disconstraint and greater persistence of alcohol related behaviors has been previously shown [50]. Disconstraint scale reflects the extent to which behavior can be constrained by future consequences [101]. Moreover, the Disconstraint scale involves measures of offending attitudes and norm violation [52], and it was found associated with non-violent delinquency in adolescence [102,103]. In this study, drivers with higher scores of Disconstraint were more likely classified as members of the DD group. This result showed the association between Disconstraint and drunken driving, suggesting that the evaluation of this personality trait may be useful in detecting potentially unsafe drinker drivers.

Conversely, participants with higher scores of Psychoticism, Negative-Emotionality, and Introversion were more likely classified in the group of controls. Previous studies showed associations between Negative-Emotionality and drinking behavior [51,104].

Notwithstanding, Negative-Emotionality is considered a measure of the sensitivity of the danger detection and anxiety and was found associated with risk-avoidance and inhibition [48,52]. Considering the above, the results presented here showed that drivers with a low level of Negative-Emotionality—characterized by poor behavioral inhibition and low risk-aversion—are prone to drive under the effect of alcohol. Similarly, lower scores of Introversion were found more likely distinctive of DD drivers. The introversion scale assesses the tendency to which people are focused on their feeling, mood, and thoughts and reflects low sociability [52]. Previous studies have discussed the association between Introversion and both avoidance behaviors [105] and inhibition [48]. Results presented here showed that low scores in this scale—being associated with low behavioral control—predicted the membership in the DD group. This result potentially suggests that the low behavioral control may act as a risk factor of the likelihood of being engaged in drinking and driving behaviors and being caught for DUI violation among drinkers. The last two results revealed that the pattern of internalizing traits could be considered a marker of safe driving habits, suggesting the usefulness of both Negative-Emotionality and Introversion personality scales in discriminating the psychological fitness-to-drive among DD. In our opinion, both low Introversion and low Negative-Emotionality are: (a) relevant markers of recidivism in drinker drivers with an history of driving under the influence; and (b) more tentatively, a sign of the risk of being caught for drunken driving. Furthermore, it cannot be ruled out that the lower scores in Psychoticism, Negative-Emotionality, and Introversion observed in the DD group could be partially attributable to the lower desirability of signs expressed by the items of these scales compared with those of the DISC personality scale. The items included in PSYC, NEGE, and INTR scales in many cases can be referred to symptoms that are clearly recognizable even by unexperienced people [106,107]. It is plausible to assume that these scales are more likely subjected to self-deception/impression management than the DISC scale. On the other hand, it cannot be excluded an effect of response management even for the DISC scale. It is likely that for items of the DISC scale the impression management acted toward an inverse and positive direction (in contrast to the other PSY-5 scales); indeed, the items of the DISC scale could be interpreted as indicative of alertness, readiness, and quickness of movements and thoughts and in turn as desirable behavioral characteristics, particularly for drivers who are submitted to reassessments of the fitness-to-drive.

Overall, this study addressed the integration of different psychological dimensions for the psychological assessment of drinker drivers. The results of the present study are limited to (a) a sample of male drivers forced to pass the visit to regain their driving license, limited to (b) DD drivers stopped with a BAC level equal or greater than 0.8 g/L and evaluated far away from the episode of intoxication. Moreover, in the present study no information was collected on the history of alcohol abuse. Further research could offer different theoretical and methodological improvements. First, the inclusion of a balanced group of female drivers could be helpful in controlling gender-related effects on psychological driving-related measures. In this regard, it cannot be overlooked that the number of men who ask to be re-authorized to drive is enormously higher than the number of women, at least in the cultural context of the present study. Furthermore, a stringent study on the long-term effects of alcohol use by manipulating the interval between the moment of intoxication and that of the assessment would be of great interest and importance. The inclusion of a non-teetotal control group of drivers would allow the study to have a greater impact with more representative results.

Finally, further research needs to be conducted to extend this investigation by comparing the performance of occasional and habitual drivers under the influence of alcohol and other substances, which would also include new variables such as attitudes toward drunken driving, general values, road traffic culture, and behavioral habits.

## 5. Conclusions

The present study provided suggestions on the suitability of widely used standardized measures of cognition, personality, and complex tasks connected to driving in discriminating DD in a sober state from non-drinker drivers.

The assessment of psychological fitness-to-drive in DD is a relevant issue for driving safety challenging the professionals of mobility centers. Previous research investigated separately different psychological dimensions (i.e., cognition, behavior, personality, and demographics) characterizing DD and rarely integrating them to reach a comprehensive and accurate protocol of evaluation. The present study focused exclusively on laboratory tests assessing psychological aspects of fitness-to-drive including cognition, driving behavior, and personality measures. The results suggested that mental rotation abilities and the resilience of attention together with some pathological personality indicators (i.e., Psychoticism, Disconstraint; Negative Emotionality; Introversion) are useful measures in identifying drivers caught for violations and probably differentially subjected to impression management or self-deception. Despite the results presented here, and the importance of personality in affecting driving behaviors, self-reported personality measures should be considered with caution [108] considering their exposure to faking good, especially in forensic assessments in which they could potentially lead to paradoxical effects. Furthermore, these results may be a relevant source of knowledge for researchers dealing with both the prediction of motor vehicle-crashes and the driver’s liability in such events [109], providing information about variables that are able in detecting drinker drivers.

This study suggests that the comprehensive evaluation of all the dimensions influencing the psychological fitness-to-drive approach, as an intervention for drunken driving, can be improved with a few, but significant, changes to the current protocols.

## Figures and Tables

**Table 1:** Parameters for the final binomial logistic regression model. Regression coefficient, standard error, z-value, Wald *χ*^2^, degrees of freedom and related *p*-value, odd ratio and 95% confidence interval are reported for each predictor included in the final model.

								95% Confidence Interval	
	B	SE	Z	Wald *χ*^2^	Df	*p*	OR	Lower	Upper
MoCA	0.1580	0.0888	1.78	3164	1	0.075	1.171	0.984	1.394
MRT	−0.0679	0.0278	−2.45	5980	1	0.014	0.934	0.885	0.987
DT	−0.0221	0.0108	−2.04	4164	1	0.041	0.978	0.958	0.999
PSYC	−0.0630	0.0313	−2.01	4052	1	0.044	0.939	0.883	0.998
DISC	0.1153	0.0255	4.53	20,509	1	<0.001	1.122	1.068	1.180
NEGE	−0.1303	0.0340	−3.84	14,713	1	<0.001	0.878	0.821	0.938
INTR	−0.0927	0.0253	−3.67	13,469	1	<0.001	0.911	0.867	0.958

## Data Availability

This study had been preregistered on Open Science Framework–10.17605/OSF.IO/BVH8K, registration form https://osf.io/ywvg4.

## Supplementary Materials

The following are available online at https://www.mdpi.com/article/10.3390/ijerph182312828/s1, Tables S1 and S2: Correlation matrix between the variables. Mean (M) and Standard Deviation (SD) for each variable in each group. * *p* < 0.05, ** *p* < 0.01, *** *p* < 0.001. AGE = age in years; EDU = years of education; MOCA = MoCA corrected score; MRT = Mental Rotation Test; OPT = Object-Perspective Taking Test; AGGR = Aggression; PSYC = Psychoticism; DISC = Discontraint; NEGE = Negative Emotionality; INTR = Introversion; DT = Schuhfried Vienna “Determination Test” score; MS = Schuhfried Vienna “Motor Speed”; RS =Schuhfried Vienna “Reaction Speed”; ATAVT = Schuhfried Vienna “Perceptual speed”.

## References

[1] National Center for Statistics and Analysis (2020). Overview of Motor Vehicle Crashes in 2019. (2020, December). (Traffic Safety Facts Research Note. Report No. DOT HS 813 060).

[2] Calinescu T., Adminaite D. (2018). Progress in Reducing Drink Driving in Europe.

[3] Jones A.W. (2014). Effects of Alcohol on Fitness to Drive. Handb. Forensic Med..

[4] Schmitz A.R., Goldim J.R., Guimarães L.S., Lopes F.M., Kessler F., Sousa T., Gonçalves V.M., Pechansky F. (2014). Factors Associated with Recurrence of Alcohol-Related Traffic Violations in Southern Brazil. Braz. J. Psychiat..

[5] Piccardi L., Palmiero M., Guariglia P., Dacquino C., Cordellieri P., Giannini A.M. (2021). Is the Risk Behaviour Related to the Ordinary Driving Violations?. Psychol. Studies.

[6] Appenzeller B.M., Schneider S., Yegles M., Maul A., Wennig R. (2005). Drugs and Chronic Alcohol Abuse in Drivers. Forensic Sci. Int..

[7] Istat (2020). Rilevazione Degli Incidenti Stradali Con Lesioni a Persone—Periodo di Riferimento: Anno 2019.

[8] Bartholow B.D., Pearson M., Sher K.J., Wieman L.C., Fabiani M., Gratton G. (2003). Effects of Alcohol Consumption and Alcohol Susceptibility on Cognition: A Psychophysiological Examination. Biol. Psychol..

[9] Sabia S., Elbaz A., Britton A., Bell S., Dugravot A., Shipley M., Kivimaki M., Singh-Manoux A. (2014). Alcohol Consumption and Cognitive Decline in Early Old Age. Neurology.

[10] Jongen S., Vuurman E.F., Ramaekers J.G., Vermeeren A. (2016). The Sensitivity of Laboratory Tests Assessing Driving Related Skills to Dose-Related Impairment of Alcohol: A Literature Review. Accid. Anal. Prev..

[11] Steele C.M., Josephs R.A. (1988). Drinking Your Troubles Away: 2. An Attention-Allocation Model of Alcohol’s Effects on Psychological Stress. J. Abnorm. Psychol..

[12] Sayette M.A., Leonard K.E., Blane H.T. (1999). Cognitive Theory and Research. Psychological Theories of Drinking and Alcoholism.

[13] Vogel-Sprott M., Easdon C., Fillmore M., Finn P., Justus A. (2001). Alcohol and behavioral control: Cognitive and Neural Mechanisms. Alcohol. Clin. Exp. Res..

[14] Curtin J.J., Fairchild B.A. (2003). Alcohol and Cognitive Control: Implications for Regulation of Behavior during Response Conflict. J. Abnorm. Psychol..

[15] Harrison E.L., Fillmore M.T. (2011). Alcohol and Distraction Interact to Impair Driving Performance. Drug Alcohol. Depend.

[16] Day A.M., Kahler C.W., Ahern D.C., Clark U.S. (2015). Executive Functioning in Alcohol Use Studies: A Brief Review of Findings and Challenges in Assessment. Curr. Drug Abus. Rev..

[17] Giancola P.R., Moss H.B., Galanter M. (1998). Executive Cognitive Functioning in Alcohol Use Disorders. Recent Developments in Alcoholism: Volume 14. The Consequences of Alcoholism.

[18] Bates M.E., Bowden S.C., Barry D. (2002). Neurocognitive Impairment Associated with Alcohol Use Disorders: Implications for Treatment. Exp. Clin. Psychopharmacol..

[19] Ledger S., Bennett J.M., Chekaluk E., Batchelor J. (2019). Cognitive Function and driving: Important for Young and Old Alike. Transp. Res. Part F.

[20] Tinella L., Lopez A., Caffò A.O., Grattagliano I., Bosco A. (2020). Spatial Mental Transformation Skills Discriminate Fitness to Drive in Young and Old Adults. Front. Psychol..

[21] Ledger S., Bennett J.M., Chekaluk E., Batchelor J., Di Meco A. (2019). Cognitive Function and Driving in Middle Adulthood: Does Age Matter?. Transp. Res. Part. F.

[22] Kwok J.C.W., Gélinas I., Benoit D., Chilingaryan G. (2015). Predictive Validity of the Montreal Cognitive Assessment (MoCA) as a Screening Tool for On-Road Driving Performance. Br. J. Occup. Ther..

[23] Nasreddine Z.S., Phillips N.A., Bédirian V., Charbonneau S., Whitehead V., Collin I., Cummings J.L., Chertkow H. (2005). The Montreal Cognitive Assessment, MoCA: A Brief Screening Tool for Mild Cognitive Impairment. J. Am. Geriatr. Soc..

[24] Folstein M.F., Robins L.N., Helzer J.E. (1983). The Mini-Mental State Examination. Arch. Gen. Psychiatry.

[25] Tavares-Júnior J., de Souza A., Alves G.S., Bonfadini J.C., Siqueira-Neto J.I., Braga-Neto P. (2019). Cognitive Assessment Tools for Screening Older Adults With Low Levels of Education: A Critical Review. Front. Psychiatry.

[26] Tinella L., Lopez A., Caffò A.O., Nardulli F., Grattagliano I., Bosco A. (2021). Cognitive Efficiency and Fitness-to-Drive along the Lifespan: The Mediation Effect of Visuospatial Transformations. Brain Sci..

[27] Alarcon R., Nalpas B., Pelletier S., Perney P. (2015). MoCA as a Screening Tool of Neuropsychological Deficits in Alcohol-Dependent Patients. Alcohol. Clin. Exp. Res..

[28] Pelletier S., Nalpas B., Alarcon R., Rigole H., Perney P. (2016). Investigation of Cognitive Improvement in Alcohol-Dependent Inpatients Using the Montreal Cognitive Assessment (MoCA) Score. J. Addict..

[29] Ney M., Tangri N., Dobbs B., Bajaj J., Rolfson D., Ma M., Ferguson T., Bhardwaj P., Bailey R.J., Abraldes J. (2018). Predicting Hepatic Encephalopathy-Related Hospitalizations Using a Composite Assessment of Cognitive Impairment and Frailty in 355 Patients With Cirrhosis. Am. J. Gastroenterol..

[30] Beatty W.W., Hames K.A., Blanco C.R., Nixon S.J., Tivis L.J. (1996). Visuospatial Perception, Construction and Memory in Alcoholism. J. Stud. Alcohol.

[31] Kunishige M., Fukuda H., Iida T., Kawabata N., Ishizuki C., MIyaguchi H. (2019). Spatial Navigation Ability and Gaze Switching in Older Drivers: A Driving Simulator Study. Hong Kong J. Occ. Ther..

[32] Kunishige M., Miyaguchi H., Fukuda H., Iida T., Nami K., Ishizuki C. (2020). Spatial Navigation Ability is Associated with the Assessment of Smoothness of Driving during Changing Lanes in Older Drivers. J. Physiol. Anthropol..

[33] Nori R., Palmiero M., Bocchi A., Giannini A.M., Piccardi L. (2020). The Specific Role of Spatial Orientation Skills in Predicting Driving Behaviour. Transp. Res. Part. F Traffic Psychol. Behav..

[34] Jornet-Gibert M., Gallardo-Pujol D., Suso C., Andrés-Pueyo A. (2013). Attitudes Do Matter: The Role of Attitudes and Personality in DUI Offenders. Accid. Anal. Prev..

[35] McMillen D.L., Adams M.S., Wells-Parker E., Pang M.G., Anderson B.J. (1992). Personality Traits and Behaviors of Alcohol-Impaired Drivers: A Comparison of First and Multiple Offenders. Addict. Behav..

[36] Cheng A.S.K., Ng T.C.K., Lee H.C. (2012). Impulsive Personality and Risk-Taking Behavior in Motorcycle Traffic Offenders: A Matched Controlled Study. Personal. Individ. Differ..

[37] Scott C., Corbin W.R. (2014). Influence of Sensation Seeking on Response to Alcohol Versus Placebo: Implications for the Acquired Preparedness Model. J. Stud. Alcohol. Drugs..

[38] Zicat E., Bennett J.M., Chekaluk E., Batchelor J. (2018). Cognitive Function and Young Drivers: The Relationship between Driving, Attitudes, Personality and Cognition. Transp. Res. Part F Traffic Psychol. Behav..

[39] Dahlen E.R., Martin R.C., Ragan K., Kuhlman M.M. (2005). Driving Anger, Sensation Seeking, Impulsiveness, and Boredom Proneness in the Prediction of Unsafe Driving. Accid. Anal. Prev..

[40] Tinella L., Caffò A.O., Lopez A., Grattagliano I., Bosco A. (2021). The Impact of Two MMPI-2-Based Models of Personality in Predicting Driving Behavior. Can Demographic Variables Be Disregarded?. Brain Sci..

[41] Machin M.A., Sankey K.S. (2008). Relationships between Young Drivers’ Personality Characteristics, Risk Perceptions, and Driving Behaviour. Accid. Anal. Prev..

[42] Zakletskaia L.I., Mundt M.P., Balousek S.L., Wilson E.L., Fleming M.F. (2009). Alcohol-Impaired Driving Behavior and Sensation-Seeking Disposition in a College Population Receiving Routine Care at Campus Health Services Centers. Accid. Anal. Prev..

[43] Jonah B.A., Thiessen R., Au-Yeung E. (2001). Sensation Seeking, Risky Driving and Behavioral Adaptation. Accid. Anal. Prev..

[44] Schell T.L., Chan K.S., Morral A.R. (2006). Predicting DUI Recidivism: Personality, Attitudinal, and Behavioral Risk Factors. Drug Alcohol Depend.

[45] Ulleberg P., Rundmo T. (2003). Personality, Attitudes and Risk Perception as Predictors of Risky Driving Behaviour among Young Drivers. Saf. Sci..

[46] Mohammadi M., Alavi S.P., Jannatifard F.P., Mohammadi Kalhory S.P., Babareisi M.P., Khodakarami R. (2015). Bipolar Disorders in Truck Drivers with Driving Licence Gategory D&C. Tehran: 5th Congress of Iranian Psychological As-sociation. Contemp. Psychol. Biann. J. Iran. Psychol. Assoc..

[47] Alavi S.S., Mohammadi M.R., Souri H., Mohammadi Kalhori S., Jannatifard F., Sepahbodi G. (2017). Personality, Driving Behavior and Mental Disorders Factors as Predictors of Road Traffic Accidents Based on Logistic Regression. Iran J. Med. Sci..

[48] Harkness A.R., Finn J.A., McNulty J.L., Shields S.M. (2012). The Personality Psychopathology—Five (PSY–5): Recent Constructive Replication and Assessment Literature Review. Psychol. Asses..

[49] Harkness A.R., Butcher J.N. (2009). Theory and Measurement of Personality Traits. Ojcford Handbook of Personality Assessment.

[50] Chassin L., Pitts S., Prost J. (2002). Binge Drinking Trajectories from Adolescence to Emerging Adulthood in a High-Risk Sample: Predictors and Substance Abuse Outcomes. J. Consult. Clin. Psychol..

[51] Elkins I.J., King S.M., McGue M., Iacono W.G. (2006). Personality Traits and the Development of Nicotine, Alcohol, and Illicit Drug Disorders: Prospective Links from Adolescence to Young Adulthood. J. Abnorm. Psychol..

[52] Magallón-Neri E., Díaz R., Forns M., Goti J., Castro-Fornieles J. (2015). Personality Psychopathology, Drug Use and Psychological Symptoms in Adolescents with Substance Use Disorders and Community Controls. PeerJ.

[53] Fine B.J. (1963). Introversion-Extraversion and Motor Vehicle Driver Behavior. Percept. Mot. Skills.

[54] Mills J.F., Kroner D.G. (2006). Impression Management and Self-Report among Violent Offenders. J. Interpers. Violence.

[55] Martino V., Grattagliano I., Bosco A., Massaro Y., Lisi A., Campobasso F., Marchitelli M.A., Catanesi R. (2015). A New Index for the MMPI-2 Test for Detecting Dissimulation in Forensic Evaluations: A Pilot Study. J. Forensic Sci..

[56] Bosco A., Massaro Y., Lisi A., Di Conza A., Campobasso F., Caffò A.O., Catanesi R., Martino V., Mazzotta N., Grattagliano I. (2020). Detecting Faking Good in Military Enlistment Procedure according to a New Index for the MMPI-2. Ital. J. Criminol..

[57] Hathaway S.R., McKinley J.C. (1942). A Multiphasic Personality Schedule (Minnesota): III. The Measurement of Symptomatic Depression. J. Psychol..

[58] Rogers R.E. (2008). Clinical Assessment of Malingering and Deception.

[59] Hathaway S.R., McKinley J.C., Butcher J.N. (1989). MMPI-2, Minnesota Multiphasic Personality Inventory-2: User’s Guide. National Computer Systems. https://www.amazon.com/MMPI-2-Minnesota-multiphasic-personality-inventory-2/dp/B0006ETD7Q.

[60] Gough H.G. (1950). The F Minus K Dissimulation Index for the Minnesota Multiphasic Personality Inventory. J. Consult. Psychol..

[61] Greene R.L. (1988). The Relative Efficacy of F-K and the Obvious and Subtle Scales to Detect Overreporting of Psychopathology on the MMPI. J. Clin. Psychol..

[62] Carson R.C., Butcher J.N. (1969). Interpretative Manual to the MMPI. MMPI: Research, Development and Clinical Applications.

[63] Lajunen T., Summala H. (2003). Can We Trust Self-Reports of Driving? Effects of Impression Management on Driver Behaviour Questionnaire Responses. Transp. Res. Part F Traffic Psychol. Behav..

[64] Lajunen T., Corry A., Summala H., Hartley L. (1997). Impression Management and Self-Deception in Traffic Behaviour Inventories. Personal. Individ. Differ..

[65] Moskowitz H., Fiorentino D. (2000). A Review of the Literature on the Effects of Low Doses of Alcohol on Driving-Related Skills.

[66] Caffò A.O., Tinella L., Lopez A., Spano G., Massaro Y., Lisi A., Stasolla F., Catanesi R., Nardulli F., Grattagliano I. (2020). The Drives for Driving Simulation: A Scientometric Analysis and a Selective Review of Reviews on Simulated Driving Research. Front. Psychol..

[67] Edwards J.D., Vance D.E., Wadley V.G., Cissell G.M., Roenker D.L., Ball K.K. (2005). Reliability and Validity of Useful Field of View Test Scores as Administered by Personal Computer. J. Clin. Exp. Neuropsychol..

[68] Jongen S., Perrier J., Vuurman E.F., Ramaekers J.G., Vermeeren A. (2015). Sensitivity and Validity of Psychometric Tests for Assessing Driving Impairment: Effects of Sleep Deprivation. PLoS ONE.

[69] Colarusso R.P., Hammill D.D. (1972). Motor-Free Visual Perception Test.

[70] Calhoun V.D., Altschul D., McGinty V., Shih R., Scott D., Sears E., Pearlson G.D. (2004). Alcohol Intoxication Effects on Visual Perception: An fMRI Study. Hum. Brain Mapp..

[71] Kaszniak A.W., Keyl P.M., Albert M.S. (1991). Dementia and the Older Driver. Hum. Factors.

[72] Keyl P.M., Rebok G.W., Gallo J.J. (1997). Screening Elderly Drivers in General Medical Settings: Toward Development of a Feasible Assessment Procedure (No. Final Report).

[73] Nechtelberger M., Vlasak T., Senft B., Nechtelberger A., Barth A. (2020). Assessing Psychological Fitness to Drive for Intoxicated Drivers: Relationships of Cognitive Abilities, Fluid Intelligence, and Personality Traits. Front. Psychol..

[74] Schuhfried GmbH (2016). Manual: Fitness to Drive Screening. Test Label DRIVESC.

[75] Harkness A.R., McNulty J.L., Ben-Porath Y.S. (1995). The Personality Psychopathology Five (PSY-5): Constructs and MMPI-2 Scales. Psychol. Asses..

[76] Elander J., West R., French D. (1993). Behavioral Correlates of Individual Differences in Road-Traffic Crash Risk: An Examination of Methods and Findings. Psychol. Bull..

[77] Martí-Belda Bertolín A., Pastor Soriano J.C., Montoro González L., Bosó Seguí P., Roca J. (2019). Persistent Traffic Offenders. Alcohol Consumption and Personality as Predictors of Driving Disqualification. Eur. J. Psychol. Appl. Leg. Context.

[78] McKnight A.J., Langston E.A., McKnight A.S., Resnick J.A., Lange J.E. (1995). Why People Drink and Drive: The Bases of Drinking-and-Driving Decisions.

[79] Sekhon J.S. (2011). Multivariate and Propensity Score Matching Software with Automated Balance Optimization: The Matching Package for R. J. Stat. Softw..

[80] Agresti (2007). An Introduction to Categorical Data Analysis.

[81] Bosco A., Spano G., Caffò A.O., Lopez A., Grattagliano I., Saracino G., Pinto K., Hoogeveen F., Lancioni G.E. (2017). Italians Do It Worse. Montreal Cognitive Assessment (MoCA) Optimal Cut-Off Scores for People with Probable Alzheimer’s Disease and with Probable Cognitive Impairment. Aging Clin. Exp. Res..

[82] Bosco A., Caffò A.O., Spano G., Lopez A. (2020). Beyond the Cutoffs: A Bayesian Approach to the Use of the Montreal Cognitive Assessment as a Screening Tool for Mild Cognitive Impairment and Dementia. Diagnosis and Management in Dementia.

[83] Vandenberg S.G., Kuse A.R. (1978). Mental Rotations, a Group Test of Three-Dimensional Spatial Visualization. Percept. Mot. Skills.

[84] Kozhevnikov M., Hegarty M. (2001). A Dissociation between Object Manipulation Spatial Ability and Spatial Orientation Ability. Mem. Cogn..

[85] Hegarty M., Waller D. (2004). A Dissociation between Mental Rotation and Perspective-Taking Spatial Abilities. Intelligence.

[86] Butcher J.N., Dahlstrom W.G., Graham J.R., Tellegen A.M., Kaemmer B. (1989). Minnesota Multiphasic Personality Inventory-2 (MMPI-2): Manual for Administration and Scoring.

[87] Hathaway S.R., McKinley J.C., Pancheri P., Sirigatti S. (1995). MMPI-2: Minnesota Multiphasic Personality Inventory-2: Manuale.

[88] IBM Corp (2012). IBM SPSS Statistics for Windows.

[89] (2019). The Jamovi Project (Version 1.0.7.0) Computer Software. https://www.jamovi.org.

[90] Hindmarch I., Bhatti J.Z., Starmer G.A., Mascord D.J., Kerr J.S., Sherwood N. (1992). The Effects of Alcohol on the Cognitive Function of Males and Females and on Skills Relating to Car Driving. Hum. Psychopharmacol. Clin. Exp..

[91] Gauthier I., Hayward W.G., Tarr M.J., Anderson A.W., Skudlarski P., Gore J.C. (2002). BOLD Activity during Mental Rotation and Viewpoint-Dependent Object Recognition. Neuron.

[92] Buckley J., Canty D., Seery N. (2018). Spatial Working Memory in Mental Rotations: A Case for Exploring Neural Efficiency and Cognitive Strategies. Proceedings of ASEE Engineering Design Graphics Division 72 Mid-Year Conference.

[93] Noël X., Sferrazza R., Van der Linden M., Paternot J., Verhas M., Hanak C., Pelc I., Verbanck P. (2002). Contribution of Frontal Cerebral Blood Flow Measured by 99mTc-Bicisate SPECT and Executive Function Deficits to Prediction Treatment Outcome in Alcohol-Dependent Patients. Alcohol. Alcohol..

[94] Ramchandani V.A., Stangl B.L., Blaine S.K., Plawecki M.H., Schwandt M.L., Kwako L.E., Sinha R., Cyders M.A., O’Connor S., Zakhari S. (2018). Stress Vulnerability and Alcohol Use and Consequences: From Human Laboratory Studies to Clinical Outcomes. Alcohol.

[95] Sinha R., Fox H.C., Hong K.I.A., Hansen J., Tuit K., Kreek M.J. (2011). Effects of Adrenal Sensitivity, Stress-and Cue-Induced Craving, and Anxiety on Subsequent Alcohol Relapse and Treatment Outcomes. Arch. Gen. Psychiatry.

[96] Enoch M.A. (2011). The Role of Early Life Stress as a Predictor for Alcohol and Drug Dependence. Psychopharmacology.

[97] Schwandt M.L., Heilig M., Hommer D.W., George D.T., Ramchandani V.A. (2013). Childhood Trauma Exposure and Alcohol Dependence Severity in Adulthood: Mediation by Emotional Abuse Severity and Neuroticism. Alcohol. Clin. Exp. Res..

[98] Stangl B.L., Vatsalya V., Zametkin M.R., Cooke M.E., Plawecki M.H., O’Connor S., Ramchandani V.A. (2017). Exposure Response Relationships during Free-Access Intravenous Alcohol Self-Administration in Nondependent Drinkers: Influence of Alcohol Expectancies and Impulsivity. Int. J. Neuropsychopharmacol..

[99] Becker H.C. (2012). Effects of Alcohol Dependence and Withdrawal on Stress Responsiveness and Alcohol Consumption. Alcohol Res..

[100] Mathias J., Lucas L.K. (2009). Cognitive Predictors of Unsafe Driving in Older Drivers: A Meta-Analysis. Int. Psychogeriatr..

[101] McNulty J.L., Harkness A.R., de Raad B., Perugini M. (2002). The MMPI-2 Personality Psychophatology-Five (PSY-5) Scales and the Five Factor Model. Big five Assessment.

[102] Veltri C.O., Graham J.R., Sellbom M., Ben-Porath Y.S., Forbey J.D., O’Connell C., Rogers R., White R.S. (2009). Correlates of MMPI-a Scales in Acute Psychiatric and Forensic Samples. J. Pers. Assess..

[103] Veltri C.O., Sellbom M., Graham J.R., Ben-Porath Y.S., Forbey J.D., White R.S. (2014). Distinguishing Personality Psychopathology Five (PSY-5) Characteristics Associated with Violent and Nonviolent Juvenile Delinquency. J. Pers. Assess..

[104] Woicik P.A., Stewart S.H., Phil R.O., Conrod P.J. (2009). The Substance Use Risk Scale: A Scale Measuring Traits Linked to Reinforcement-Specific Substance Use Profiles. Addict. Behav..

[105] Walker D.L. (2020). Extraversion–Introversion. The Wiley Encyclopedia of Personality and Individual Differences: Models and Theories.

[106] Edwards A.L. (1957). The Social Desirability Variable in Personality Assessment and Research.

[107] Lane R.D., Meringas K.R., Schwartz G.E., Huang S.S., Prusoff B.A. (1990). Inverse Relationship between Defensiveness and Lifetime Prevalence of Psychiatric Disorder. Am. J. Psychiatry.

[108] Monaro M., Mazza C., Colasanti M., Ferracuti S., Orrù G., di Domenico A., Sartori G., Roma P. (2021). Detecting Faking-Good Response Style in Personality Questionnaires with Four Choice Alternatives. Psychol. Res..

[109] Sanjurjo-de-No A., Arenas-Ramírez B., Mira J., Aparicio-Izquierdo F. (2021). Driver Liability Assessment in Vehicle Collisions in Spain. Int. J. Environ. Res. Public Health.

