# Supplementary material for: Reassessing Fitness-to-Drive in Drinker Drivers: The Role of Cognition and Personality
Source: Int J Environ Res Public Health. 2021 Dec 5;18(23):12828. doi: 10.3390/ijerph182312828 (PMC8657624; doi:10.3390/ijerph182312828)
Supplement: Supplementary file 1 [file ijerph-18-12828-s001.zip › Table_S1.pdf]

Table S1. Expected sensitivity in discriminating participants' groups.

	expected discriminatory efficiency		
Variables	low		high
MoCA	✓		
MRT			✓
OPT			✓
DT			✓
RS			✓
MS			✓
ATAVT			✓
AGGR	✓		
PSYCH		–	
DISC	✓		
NEGE	✓		
INTR	✓		
L			✓
F-K			✓

AGE=age in years; EDU=years of education; MOCA=MoCA corrected score; MRT=Mental Rotation Test; OPT= Object-Perspective Taking Test; AGGR=Aggression; PSYC=Psychoticism; DISC=Discontraint; NEGE=Negative Emotionality; INTR=Introversion; L=Lie; F-K=Gough Dissimulation Index; DT=Schuhfried Vienna "Determination Test" score; MS=Schuhfried Vienna "Motor Speed"; RS=Schuhfried Vienna "Reaction Speed"; ATAVT=Schuhfried Vienna "Perceptual speed". —= no expected results.
