# Supplementary material for: Reassessing Fitness-to-Drive in Drinker Drivers: The Role of Cognition and Personality
Source: Int J Environ Res Public Health. 2021 Dec 5;18(23):12828. doi: 10.3390/ijerph182312828 (PMC8657624; doi:10.3390/ijerph182312828)
Supplement: Supplementary file 1 [file ijerph-18-12828-s001.zip › Table_S2.pdf]

Table S2. Correlation matrix between the variables. Mean (M) and Standard Deviation (SD) for each variable in each group. *p< 0.05, **p< 0.01, ***p< 0.001. Reliability coefficients are reported in diagonal. AGE=age in years; EDU=years of education; MOCA=MoCA corrected score; MRT=Mental Rotation Test; OPT= Object-Perspective Taking Test; AGGR=Aggression; PSYC=Psychoticism; DISC=Discontraint; NEGE=Negative Emotionality; INTR=Introversion; L=Lie; F-K=Gough Dissimulation Index ;DT=Schuhfried Vienna “Determination Test” score; MS=Schuhfried Vienna “Motor Speed”; RS=Schuhfried Vienna “Reaction Speed”; ATAVT=Schuhfried Vienna “Perceptual speed”.

	AGE	EDU	MoCA	MRT	OPT	DT	RS	MS	ATAVT	AGGR	PSYC	DISC	NEGE	INTR	L	F-K		No drinker drivers (N=90)		Drinker drivers (N=90)	
																		M	sd	M	sd
AGE	—																	38.2	17.9	38.2	13.6
EDU	-0.361***	—																11.7	2.64	11.3	3.54
MoCA	-0.296***	0.369***	0.688															24.6	3.20	24.9	2.70
MRT	-0.418***	0.300***	0.420***	0.802														20.2	9.80	15.9	8.23
OPT	0.406***	-0.450***	-0.467***	-0.564***	0.639													72.2	58.0	81.5	50.4
DT	-0.605***	0.345***	0.469***	0.477***	-0.444***	0.959												66.4	25.4	61.9	22.4
RS	-0.345***	0.153*	0.257***	0.308***	-0.261***	0.453***	0.975											56.0	27.5	46.0	23.5
MS	-0.381***	0.206**	0.230**	0.254***	-0.311***	0.388***	0.398***	0.980										57.5	25.2	53.8	21.1
ATAVT	-0.425***	0.311***	0.409***	0.335***	-0.433***	0.479***	0.276***	0.282***	0.630									53.2	30.4	51.3	27.6
AGGR	-0.120	0.037	0.050	0.057	-0.086	0.081	0.106	0.031	-0.022	0.615								52.8	10.5	54.1	11.6
PSYC	-0.025	-0.116	-0.134	0.024	0.058	-0.049	0.050	-0.016	-0.118	0.320***	0.606							57.2	8.26	52.1	10.5
DISC	-0.251***	0.020	0.083	0.061	-0.067	0.171*	0.116	0.062	0.104	0.498***	0.343***	0.663						50.8	9.47	56.4	12.0
NEGE	-0.009	-0.052	-0.047	0.140	0.004	0.031	0.148*	0.001	-0.004	0.226**	0.623***	0.169*	0.693					58.0	9.43	48.6	8.34
INTR	0.051	-0.001	0.027	0.047	-0.037	-0.081	0.122	-0.008	-0.001	-0.270***	0.021	-0.196**	0.261***	0.718				52.2	9.17	44.0	8.92
L	0.112	-0.147*	-0.192*	-0.114	-0.161*	-0.264***	-0.225**	0.031	-0.130	-0.238**	-0.266***	-0.330***	-0.310***	0.013	0.604			49.1	7.43	50.0	8.93
F-K	-0.053	-0.222**	-0.209**	-0.008	0.068	0.011	0.123	-0.080	-0.041	0.232**	0.561***	0.279***	0.509***	0.034	-0.486***	—		-5.79	8.36	-8.59	7.86
GROUP	0.002	-0.061	0.036	-0.234**	0.086	-0.094	-0.192***	-0.079	-0.033	0.061	-0.264***	0.253***	-0.468***	-0.415***	0.051	-0.171*	—	—		—	
